# Parkinson's Disease-Related Biomarkers That May Appear in Amphetamine Abusers

**DOI:** 10.1155/2021/3081891

**Published:** 2021-10-19

**Authors:** Aziza Al-Rafiah, Rania Magadmi, AbdulAziz Al-Kaabi, Nimah Alsomali

**Affiliations:** ^1^Department of Medical Laboratory Technology, Faculty of Applied Medical Sciences, King Abdulaziz University, Jeddah, Saudi Arabia; ^2^Pharmacology Department, Faculty of Medicine, King Abdulaziz University, Saudi Arabia; ^3^Research Center, Neuroscience Research Department, King Fahad Medical City, Riyadh, Saudi Arabia

## Abstract

Parkinson's disease (PD) is one of the most common neurodegenerative disorders. Amphetamine addiction may cause serious of psychotic and physical damage to humans. There is some evidence that shows that amphetamine may increase the risk of PD. Thus, this study is aimed at comparing the PD serum biomarkers between amphetamine addicts and PD patients and utilizing them as diagnostic biomarkers for the early detection of PD incidence among amphetamine addicts. In the current study, nineteen amphetamine addicts, aged <40, were recruited from the Al Amal Psychiatric hospital, Jazan, Saudi Arabia. Nineteen PD patients and 19 healthy controls, who have never taken amphetamine, were also recruited. Blood samples were withdrawn from all groups. A biomarker multiplex assay from MILLIPLEX was used to assess the levels of serum amyloid-P (SAP), complement C4, C-reactive protein (CRP), and CRP/albumin ratio in serum samples (Vitros 350® slide was used to assess the albumin). All data were statistically analyzed using one-way ANOVA. The results showed that SAP and CRP levels were significantly higher in amphetamine addicts compared to healthy controls (*p* = 0.0001 and *p* = 0.0001, respectively). The results of amphetamine addicts were comparable to PD levels. However, there are no significant differences between all studied groups concerning complement C4 level. Moreover, albumin levels were significantly decreased and CRP/Albumin ratio levels were significantly increased in amphetamine addicts (*p* = 0.01 and *p* = 0.041, respectively) in contrast with controls. These findings indicate that the increased level of these inflammatory biomarkers (SAP and CRP) in the amphetamine addicts may give a potential possibility of their serum level to be used as screening markers to detect PD development in the amphetamine addict. It may be useful to evaluate the changes in easily accessible and cost-effective parameters such as the serum CRP/albumin ratio.

## 1. Introduction

Neurodegenerative disorders are a condition that results from the loss of structure or function of neurons in the central nervous system (CNS). Parkinson's disease (PD) is considered one of the worst of these debilitating and devastating disorders [1]. In 1817, neurologist Dr. James Parkinson discovered this disease and named it shaking palsy. This neurodegenerative disease is characterized by the depletion and degeneration of dopaminergic substantia nigra pars compacta (DASNc) neurons [2]. The exact etiology and pathogenesis of PD are unclear. DASNc neurons can be degenerated due to oxidative stress, excitotoxicity, mitochondrial dysfunction, or inflammatory damage [3].

Amphetamine is a synthetic drug that directly and indirectly influences the CNS and subsequently affects the peripheral organs [4]. It is the most common drug addiction in Saudi Arabia in the last two decades [5]. Amphetamine is a CNS stimulant; it appears to increase concentration, wakefulness, mood, and physical performance which lead to widespread use by students, academics, and medical professionals [6].

However, addiction of this drug can cause physical and psychological diseases like acute myocardial infarction, pulmonary edema, cerebral vasculitis, and schizophrenia [7, 8]. Among the major dangers of addiction of amphetamine drugs are the structural changes that can occur in the brain. Amphetamine addiction can destroy gray matter in the brain as well as dopamine receptors. Therefore, prolonged addiction of amphetamine is one of the risk factors for the development of PD [9].

Inflammatory and oxidative stress changes have been reported as an important biochemical mechanism mediating the addiction of these drugs [10]. There are peripheral (in serum) inflammatory biomarkers that change as a result of PD like serum amyloid-P (SAP), complement C4, and C-reactive protein (CRP) [3, 11, 12]. Thus, in the current study, we hypothesize that these inflammatory biomarkers may also change in the serum of the amphetamine addicts. Such inflammatory biomarkers will be quantified in the serum samples of both amphetamine abusers and normal healthy controls to see if there are any significant changes that occurred. Studying this correlation may help in the early diagnosis and treatment of PD in amphetamine addicts and increases the awareness about the risk of amphetamine addiction in our community.

## 2. Methods

### 2.1. Study Design and Population

This is a case control, observational study. A total of 19 healthy subjects (group I, age: 40.24 ± 5.3 years), 19 amphetamine addicts (group II, age: 32.24 ± 1.72 years), and 19 PD-confirmed patients (group III, age: 50.74 ± 1.45 years) were recruited. The inclusion criteria for group I were males, healthy subjects, aged less than 45 years old, and with no history of drug addicts or CNS disorders. The inclusion criteria for group II were males, amphetamine addicts for at least two years, and newly admitted to the hospital; they did not start the rehabilitation program yet; and they are not known to have any chronic medical conditions such as diabetes, hypertension, heart, and renal diseases. The inclusion criteria for group III were confirmed diagnosed cases with any type of PD and with no history of amphetamine addicts.

A blood sample was drawn from all subjects to measure serum SAP, CRP, and CC4 using a Luminex human neurodegenerative kit. Also, a questionnaire was distributed among group II only to collect a detailed history with regard to amphetamine addiction.

The informed consent was obtained from all of the study participants after explaining to them (or their caregiver) the aim and the procedure of the study. The study protocol was approved by the local ethics committee of King Fahad Medical City (KFMC) (IRB number 16-450, IRB registration number with KACST, KSA H-01-R-012, IRB registration number with PHRP/NIH/USA: IRB00010471, and approval number federal wide assurance NIH, USA: FWA00018774).

### 2.2. Questionnaire Design

In the current study, the questionnaire was only distributed among amphetamine addicts to correlate the history of amphetamine addiction with the expression of biomarkers and the risk of PD. The questionnaire comprised 9 questions which were divided into two parts. The first part was related to the demographic data of the participants. The second part was aimed at assessing the dose and frequency of amphetamine addiction and duration of addiction (years) and the age of the participants when they started the drug.

### 2.3. Inflammatory Biomarker Assessment (SAP, CRP, and CC4)

Human neurodegenerative disease panel 2 kit biomarkers multiplex assay from MILLIPLEX with number Cat #: HNDG2MAG-36K was used to assess the level of these biomarkers which was used to assess the levels of SAP, CRP, and CC4. Frozen serum samples, from amphetamine addicts, PD patients, and healthy subjects, were assessed for all the above parameters in duplicate at one time by using a single plate. The procedure was done according to the manufacturer assay protocols. For data analysis, a Luminex 200 machine and MILLIPLEX Analyst software were used.

The kit uses a 96-well format, containing a lyophilized standard cocktail and 2 quality controls that can measure up to 38 serum samples in duplicate. Multidimensional fatigue inventory (MFI) measurements were obtained, and data was analyzed accordingly for high sensitivity, consistency, and reproducibility.

In summary, 25 *μ*g of serum (1 : 2 diluted) was incubated with antibody-conjugated magnetic beads for overnight at a four-degree temperature inside the fridge. Bead complexes after being rinsed were kept with 50 *μ*l biotinylated detection antibody for half an hour on a plate shaker at room temperature. After that, they were incubated with 50 *μ*l streptavidin-phycoerythrin for half an hour on a plate shaker at (20-25°C). After washing 3 times, 100 *μ*l of Sheath Fluid was added to all wells. Bead complexes were then read on a Run plate on Luminex® 200™ and analyzed by MAGPIX® with xPONENT® software.

### 2.4. Measurement of Albumin in Serum

Vitros 350® albumin slides were used (lot number 0928-9243) to assess the albumin level. The serum sample does not need any preparation or dilution since all the participants' albumin level was within the system's reportable (dynamic) range. Then, the serum was added to its matched tubes, either patients or controls. After that, samples were placed on the rack in the right position, and the Vitros 350® chemistry analyzer was started. It took 8 to 10 minutes to release the results.

### 2.5. Statistical Analysis

Data were analyzed using IBM SPSS Statistical for Windows, version 21 (IBM Corp., Armonk, NY, USA). Descriptive data were expressed as mean and standard error of the mean (SEM). All data were statistically analyzed using a one-way analysis of variance (ANOVA) test. *p* values less than 0.05 were considered significant. Graphs were made by GraphPad Prism version 8 software (GraphPad Software, La Jolla, CA, USA).

## 3. Results

### 3.1. Sociodemographic Data of Amphetamine Addict Participants

Based on the questionnaire that was distributed among the amphetamine addict participants, the mean age was 32.23 ± 7.1 years as shown in Table 1. More than half of the amphetamine addict participants have only intermediate education (52.6%). Among all amphetamine addict participants, only one participant was a nonsmoker (5.3%), while 7 participants were taking amphetamine and Toombak (36.8%) and 12 were not taken (63.2%).

### 3.2. Assessment of Inflammatory Biomarkers

#### 3.2.1. Serum Amyloid-P (SAP)

**Figure 1:**
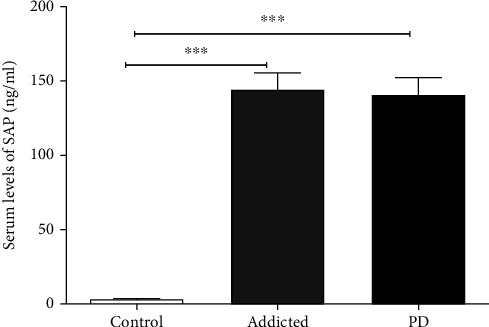
Comparison of serum amyloid protein (SAP) level between amphetamine addicts, Parkinson's disease (PD) patients, and controls. Data are expressed as mean ± standard error. Significance was made using one-way ANOVA (LSD) test. ^∗∗∗^Statistically significant at *p* < 0.0001.

In the current study, serum SAP levels in amphetamine addicts (144.29 ± 11.14 ng/ml) and PD patients (140.88 ± 11.41 ng/ml) were significantly higher compared to that in controls (3.40 ± 0.28 ng/ml) (*p* < 0.0001, for both). Meanwhile, there was a nonsignificant difference of serum SAP level between amphetamine addicts and PD patients (*p* = 0.797) as shown in Table 2 and Figure 1.

#### 3.2.2. Serum C-Reactive Protein (CRP)

In the present study, the serum CRP levels in amphetamine addicts (6.54 ± 0.63 ng/ml) and PD patients (5.46 ± 0.41 ng/ml) were significantly higher compared to that in controls (3.75 ± 0.32 ng/ml) (*p* = 0.0001 and *p* = 0.012, respectively).

**Figure 2:**
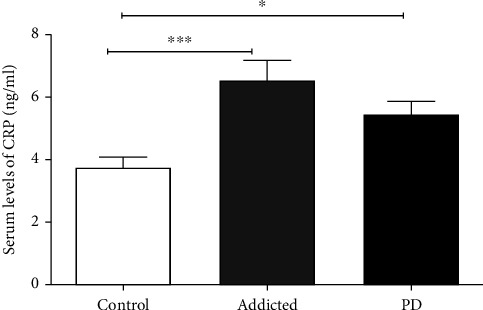
Comparison of serum C-reactive protein (CRP) level between amphetamine addicts and Parkinson's disease (PD) patients and controls. Data are expressed as mean ± standard error. Significance was made using one-way ANOVA (LSD) test. ^∗^*p* < 0.050; ^∗∗∗^*p* < 0.0001.

Meanwhile, there was a nonsignificant difference of the serum CRP level between amphetamine addicts and PD patients (*p* = 0.105) as shown in Table 2 and Figure 2.

#### 3.2.3. Serum Complement C4

**Figure 3:**
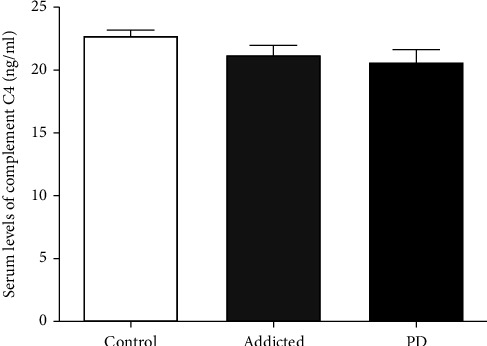
Comparison of serum complement C4 level between amphetamine addicts and PD patients and controls. Data are expressed as mean ± standard error. Significance was made using one-way ANOVA (LSD) test.

Results of this study showed that serum complement C4 levels in amphetamine addicts (21.2 ± 0.75 ng/ml) and PD patients (20.62 ± 1.01 ng/ml) were comparable to that in controls (22.73 ± 0.46 ng/ml) (*p* = 0.191 and *p* = 0.065, respectively). Besides, there was no significant difference in serum C4 level between amphetamine addicts and PD patients (*p* = 0.604), as shown in Table 2 and Figure 3.

### 3.3. Serum Albumin Level Measurement

**Figure 4:**
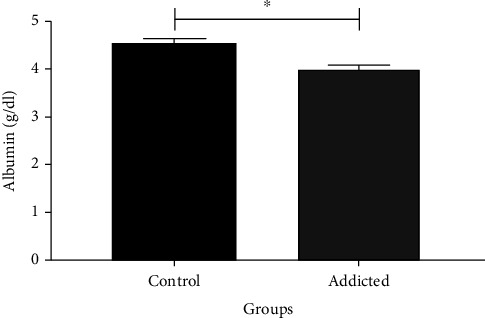
Comparison of serum albumin level between amphetamine addicts and controls. Data are expressed as mean ± standard error. Significance was made using *t*-test. ^∗^*p* < 0.050.

**Figure 5:**
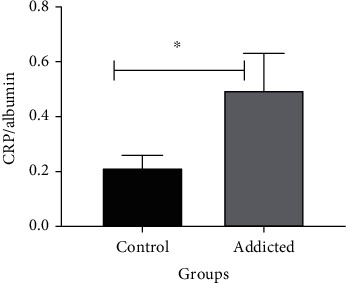
Comparison of CRP/albumin ratio between amphetamine addicts and healthy controls. Data are expressed as mean ± standard error. Significant was made using *t*-test. ^∗^*p* < 0.050.

In the current study, the albumin serum level in amphetamine addicts was significantly decreased (*p* = 0.001) compared to that in healthy controls as shown in Figure 4 and Table 3. The albumin level revealed concentrations of 4.01 ± 0.06 g/dl in the serum of amphetamine addicts and 4.57 ± 0.07 g/dl for healthy controls, while the CRP/albumin ratio in amphetamine addicts was significantly increased (*p* = 0.041) in contrast with that in controls as shown in Figure 5 and Table 3.

### 3.4. ROC Curve of Measured Parameters

**Figure 6:**
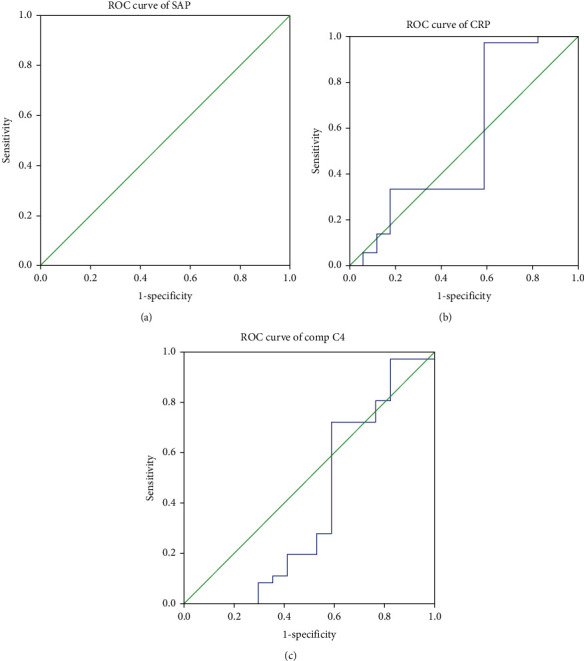
ROC curve of SAP (a), CRP (b), and Comp C4 (c).

The cutoff point of SAP was 206.00. This value was significant to diagnose disease from normal (*p* < 0.0001) with area under the curve of 1.00, standard error of 0.00, and 95% CI of 1.00-100. The cutoff point of CRP was 228.89. This value was insignificant to diagnose disease from normal (*p* = 0.529) with area under the curve of 0.554, standard error of 0.098, and 95% CI of 0.362-0.745. The cutoff point of Comp C4 was 228.89; this value was insignificant to diagnose disease from normal (*p* = 0.230) with area under the curve of 0.397, standard error of 0.098, and 95% CI of 0.204-0.590 (Figure 6).

## 4. Discussion

Although the exact cause for loss of dopamine neurons in PD remains unclear, increasing evidence indicates that the mitochondrial dysfunction, oxidative stress, and inflammation are involved in the main mechanisms of neuron loss. Many publications examined the connection between amphetamine abuse and development of PD [9, 13]. However, no previous study could find a way to detect early the amphetamine addict who is at risk of developing PD. Thus, this study was conducted to investigate well-known, noninvasive biomarkers that could be utilized to diagnose early and prevent amphetamine-induced PD.

Drug abuse is considered a public, security, and occupational problem facing the Jazan region in Saudi Arabia. There are many other names for amphetamine in Saudi Arabia as street names; the most common names are Alabyad (white), Abu mlaf, Lajah, Al qeshtah, and Al asfaar (yellow). In the last few years, there was an increase in the use of cannabis and amphetamine. The risk factors for this initiation are peer pressure and psychosocial stresses, as well as social communication media and electronic websites. All these factors fire the drug abuse and increase its distribution [14].

In the present study, it was found that people among the age group of 18–60 years (mean age 32 years) with low school education had a tendency to be more inclined to drug addiction (47.1%). Education helps people to learn skills and develop perceptions of risk. A study conducted in Copenhagen found that those with the lowest level of schooling were most frequently heavy smokers, heavy drinkers, and the most physically inactive, corroborating our findings [15].

Similarly, Ibrahim et al. (2018) conducted a retrospective study in the Psychiatric Rehabilitation Center (PRC) in Tabouk, Al-Qassim, Buraidah, one of the four main specialized facilities to treat and prevent substance use disorders in Saudi Arabia [16, 17]. The investigators stated that the maximum substance abuse in the study was found among the age group of 20–40 years (37.7%) (*p* < 0.022) with a tendency for people with high school education to be more inclined to drug addiction (41.9%). In contrast to the current study, a study in Nepal found the highest rate of drug abuse among youth ages 18 to 20 (22.7%) and 21 to 25 (21.5%). This could be explained by Koenig et al. (2014) who found that those with the least schooling were more frequently heavy smokers and drinkers [18]. These results suggest that education level has some influence on the decision to abuse drugs.

Many previous cohort studies reported the increase in the risk of PD among amphetamine addicts [9, 13, 14]. Callaghan et al. (2010) reported an increase in the incidence of PD in methamphetamine users in an epidemiological investigation based on data from California statewide hospital records. They identified 1,863 methamphetamine users, 9,315 patients hospitalized for appendicitis as a nondrug control group, and 1,720 cocaine users as a drug control group. All subjects were aged at least 50 years, had been hospitalized in California between 1990 and 2000, and had been followed for up to 10 years after discharge. The methamphetamine user group showed an elevated incidence of PD, with a 165% higher risk for the development of PD than the patients from the control group [13]. These results have been reproduced later by the same group using a larger- and more-age-diverse group of patients (40,000 people hospitalized for methamphetamine versus 200,000 for appendicitis and 35,000 for cocaine) and a 16-year follow-up period [9].

These two studies are the first to link methamphetamine addiction in young adulthood with the development of PD in middle age or later, strongly supporting that methamphetamine use increases the risk for developing PD.

To define the mechanism of amphetamine-induced PD, Granado and his colleges conducted an experimental animal study. They reported that exposure to methamphetamine damages dopaminergic fibers in the striatum and their cell bodies in the substantia nigra, echoing the degeneration pattern observed in human patients with PD. Selective damage to dopaminergic terminals in the striatum has also been observed in human methamphetamine users, although there is no evidence so far that methamphetamine damages dopaminergic cell bodies in the human SNpc [19]. Given these results, it is reasonable to think that methamphetamine use may predispose consumers to the future development of PD.

Previous studies showed that the amphetamine addicts had morphological changes in the substantia nigra that resemble PD before they showed the clinical signs of PD [9, 20]. However, these morphological changes need invasive and expensive techniques and personnel to be assessed. In the present study, the level of inflammatory biomarkers was assessed in the serum samples of amphetamine addicts and PD patients to find the connection between the appearance of these biomarkers in addicts and the risk of PD. SAP, CRP, and CC4 have been chosen in this study because they could be measured in simple, noninvasive, and inexpensive biochemical tests.

The findings of this study showed a significant increase in the SAP of amphetamine addicts compared to the controls. This increase in SAP among amphetamine addicts was in the range of PD. These results are consistent with the proteomic study of SAP applied to PD patients and normal healthy controls [3].

Moreover, Chen and his colleagues applied a proteomic strategy, by utilizing two-dimensional electrophoresis and mass spectrometry, to analyze two sample pools of plasma from the healthy individuals and PD subjects. They reported that SAP was found differentially expressed between these pools. SAP level increased by approximately 5-fold in PD samples, and the ELISA procedure revealed a significant increase in SAP concentration in the plasma of PD subjects, with a sensitivity of 94.1% and specificity of 87.5%. They concluded that there was potential feasibility of plasma SAP as a marker to approach PD.

Long-term exposure to amphetamine causes a range of cognitive deficits, which involve several mechanisms [21]. For example, amphetamine exposure has been shown to be associated with neuroinflammation in several brain areas, due to its addictive effect. These results may suggest the peripheral contribution of amphetamine addiction in the inflammatory process involving neurodegeneration in PD. However, in a cross-sectional study between PD patients and controls, the naturally occurring antibody (Nab) titer for SAP (Amyloid-beta1-42) was found to be nonsignificantly associated with the occurrence of PD [22]. Similarly, Brosseron et al. stated that there was a significant positive correlation between the SAP and the age of the subjects with the parkinsonism disease [23, 24].

In the present study, in line with the literature, the CRP level in amphetamine addicts was also significantly increased, suggesting the involvement of amphetamine addiction in the neuroinflammatory process in PD pathogenesis. Several studies which were conducted on CRP for the PD patients reconcile with the results in this study. Akıl et al. (2015) showed that CRP concentrations were significantly higher in patients with PD than in healthy controls in their study performed with 51 patients with PD and 50 healthy controls . [25]

Some of these indicate that a high CRP level strengthens the clinical evidence associated with PD which results from an inflammatory response, whereas the other showed that the baseline CRP level is associated with the risk of death of patients with PD [12]. In ischemic stroke patients, the elevation of CRP was also correlated with a bad prognosis [26]. Additionally, the CRP level was also higher in cannabis, nicotine, and alcohol dependence [27]. Also, it has been shown that CRP concentrations rise in chronic diseases such as hemorrhagic cerebrovascular disease, AD, and PD [28].

CRP has been identified as a common inflammation-related cytokine. Although publications indicate that CRP is associated with the pathogenesis of neurological disorders and deemed to be a “risk factor” for PD, studies have also demonstrated a link between CRP and chronic inflammatory and neurodegenerative diseases, such as cardiovascular disease, diabetes, stroke, and Alzheimer's disease, as well as PD [28]. Up to now, some epidemiological studies have explored CRP levels and PD risk. However, results in the literature regarding CRP levels in PD patients are still contradictory. Some studies found a significant increase in CRP levels in subjects suffering from PD compared with healthy controls [29, 30].

Unlike the SAP and CRP levels, there is no significant increased level of complement C4 in the serum of amphetamine addicts comparing with controls. In addition, the CC4 level was to be found significantly higher in chronic opioid smackers [31]. Similarly, Veselý et al. (2018) investigated whether elevation of serum inflammatory marker levels may indicate the progression of clinical impairment in PD patients [32]. In 47 PD patients, the serum levels of the C3 and C4 part of the complement and Interleukin-6 (IL-6) were measured. The results at baseline and after 2 years were correlated with scales measuring memory, depression, motor symptoms, and quality of life.

Patients with higher levels of C3 and C4 at baseline had decreased quality of life, verbal ability, and memory. Patients with higher IL-6 at baseline showed worse depression scores at 2 years. Patients with persistently higher levels of C3 and C4 at 2 years had worse quality of life and memory ability.

The results of this study showed a significant decrease in serum albumin level in amphetamine addicts in parallel with the significant increase in the CRP concentration (Figure 4). Therefore, the CRP/albumin was found to be also significantly increased (Figure 5). Albumin is considered a chemical parameter associated in oxidative stress [33].

According to our knowledge, this study is the first to assess serum SAP, CRP, and CC4 in amphetamine addicts. Generally, except for CC4, the data in this study complied with the literature. In this study, the levels of SAP and CRP were in parallel with the stated hypothesis.

## 5. Conclusions and Recommendations

This study was conducted to assess the level of inflammatory biomarkers (SAP, CRP, and CC4) in the serum samples of amphetamine abusers. These results may indicate the role of amphetamine addiction to accelerate the appearance of these biomarkers. Except for the complement C4, our results indicate the increased level of these inflammatory biomarkers in the amphetamine abusers. This may give a potential possibility of their serum level to be predictive for PD development in amphetamine addicts which can reflect on the diagnosis and therapy. However, the change in the levels of these biomarkers was not definitive to be causing neurodegenerative diseases including PD. For further studies, we recommend increasing sample numbers to obtain more validation for our results and statistical significance. Furthermore, assessing other unstudied sensitive inflammatory biomarkers and interleukins is also recommended. Finally, we need to employ more valuable, sensitive, and specific techniques and biomarkers.

## Figures and Tables

**Table 1:** Sociodemographic characteristics of the amphetamine addict participants (*n* = 19).

Parameters		Count	%
Age (years)		32.24 ± 1.72	
Gender	Male	19	100%

Education	University	4	21%
	Intermediate	5	26.4%
	Secondary	10	52.6%

Smoking	Yes	18	94.7%
	No	1	5.3%

Toombak	Yes	7	36.8%
	No	12	63.2%

**Table 2:** Comparison of measured parameters between patients and controls.

	Groups	*N*	Mean	Std. error	*p* value
SAP (ng/m)	Controls	19	3.4	0.28	—
	Amphetamine addicts	19	144.29	11.14	0.0001^∗∗∗^
	Parkinson's patients	19	140.88	11.41	0.0001^∗∗∗^

CRP (ng/m)	Controls	19	3.75	0.32	—
	Amphetamine addicts	19	6.54	0.63	0.0001^∗∗∗^
	Parkinson's patients	19	5.46	0.41	0.012^∗^

CC4 (ng/m)	Controls	19	22.73	0.46	—
	Amphetamine addicts	19	21.2	0.75	0.191
	Parkinson's patients	19	20.62	1.01	0.065

Data are expressed as mean ± standard error. ^∗^Difference between participants and controls was made using one-way ANOVA test.

**Table 3:** Comparison of serum albumin level between amphetamine addicts and healthy controls.

Group statistics					
	Groups	*N*	Mean	Std. error	*p* value
Albumin (g/dl)	Controls	19	4.57	0.07	0.001^∗∗^
	Amphetamine addicts	19	4.01	0.06	

CRP/albumin	Controls	19	0.21	0.04	0.041^∗^
	Amphetamine addicts	19	0.5	0.13	

Data are expressed as mean ± standard error. ^∗^Difference between amphetamine addicts and controls was made using *t*-test.

## Data Availability

The data used to support the findings of this study are available from the corresponding author upon request.
